# Selection of Embedding Dimension and Delay Time in Phase Space Reconstruction via Symbolic Dynamics

**DOI:** 10.3390/e23020221

**Published:** 2021-02-11

**Authors:** Mariano Matilla-García, Isidro Morales, Jose Miguel Rodríguez, Manuel Ruiz Marín

**Affiliations:** 1 Facultad de Economicas y Empresariales, Universidad Nacional de Educación a Distancia (UNED), 28050 Madrid, Spain; 2 Telefónica, 28040 Madrid, Spain; isildur20022003@yahoo.es; 3 Departamento Metodos Cuantitativos, Ciencias Juridicas y Lenguas Modernas, Universidad Politecnica de Cartagena, 30201 Cartagena, Spain; Jose.Rodriguez@upct.es (J.M.R.); manuel.ruiz@upct.es (M.R.M.)

**Keywords:** symbolic analysis, symbolic entropy, delay time selection, dynamic reconstruction

## Abstract

The modeling and prediction of chaotic time series require proper reconstruction of the state space from the available data in order to successfully estimate invariant properties of the embedded attractor. Thus, one must choose appropriate time delay $\tau^{*}$ and embedding dimension *p* for phase space reconstruction. The value of $\tau^{*}$ can be estimated from the Mutual Information, but this method is rather cumbersome computationally. Additionally, some researchers have recommended that $\tau^{*}$ should be chosen to be dependent on the embedding dimension *p* by means of an appropriate value for the time delay $\tau_{w}=\left(p-1\right)\tau^{*}$, which is the optimal time delay for independence of the time series. The C-C method, based on Correlation Integral, is a method simpler than Mutual Information and has been proposed to select optimally $\tau_{w}$ and $\tau^{*}$. In this paper, we suggest a simple method for estimating $\tau^{*}$ and $\tau_{w}$ based on symbolic analysis and symbolic entropy. As in the C-C method, $\tau^{*}$ is estimated as the first local optimal time delay and $\tau_{w}$ as the time delay for independence of the time series. The method is applied to several chaotic time series that are the base of comparison for several techniques. The numerical simulations for these systems verify that the proposed symbolic-based method is useful for practitioners and, according to the studied models, has a better performance than the C-C method for the choice of the time delay and embedding dimension. In addition, the method is applied to EEG data in order to study and compare some dynamic characteristics of brain activity under epileptic episodes

## 1. Introduction

For the theory of state space reconstruction suggested by Packard, Takens et al. [1,2] is the base for data-driven analysis and prediction of chaotic systems. It can be proved through Taken’s theorem [2] that the strange attractor of the chaotic systems could be properly recovered from only one projection of the dynamic system. The fundamental theorem of reconstruction of Takens establishes a sufficient condition (but not necessary) given by p≥2d+1, where *d* is the fractal dimension of the underlying chaotic attractor, and *p* stands for the embedding dimension used for phase space reconstruction. Nevertheless, no condition is given regarding the time delay.

A popular method for state space reconstruction is the method of delays. It consists of embedding the observed scalar time series ${\left\{X_{t}\right\}}_{t\in I}$ in one *p*-dimensional space $X_{p}^{\tau }\left(t\right)=\left(X_{t},X_{t+\tau },\cdots ,X_{t+(p-1)\tau }\right)for\;t\in I$, where τ is the time delay for the reconstruction, *p* is the embedding dimension, and *I* is a set of time indexes of cardinality *T*. Notice that the number of points inserted in the *p*-dimensional space is M=T−(p−1)τ and all dynamic properties, such as dependencies, periodicity, and complexity changes, can be extracted from it. That is, there is a differentiable homomorphism from the orbits of the chaotic attractor in the reconstructed space $R^{p}$ to the original system.

The selection of the parameters *p* and $\tau^{*}$ is a challenge. An improper choice can result in a spurious indication of a nonlinear complex structure when the system is linear. Albeit specialized literature provides different methods to select the parameters for state space reconstruction, none of them that are superior to the remaining ones in all aspects. In general, the optimal strategy for parameter selection will depend on the time series and a complexity measure (e.g., Lyapunov Exponents or Correlation Dimension). There are two different approaches to the selection of the parameters *p* and $\tau^{*}$. The first approach considers that *p* and $\tau^{*}$ are selected independently from each other. For example, the G-P algorithm for the selection of *p* proposed by Albano et al. [3] and different proposals for the selection of the time delay $\tau^{*}$ based on Mutual Information [4], autocorrelation and high-order correlations [5], filling factor [6], wavering product [7], average displacement AD [8], and multiple autocorrelation [9]. The second approach considers that the parameters *p* and $\tau^{*}$ are closely related when the time series under consideration is noisy and has finite length. A great number of experiments indicate that *p* and $\tau^{*}$ are related with the time delay for independence of the time series through $\tau_{w}=\left(p-1\right)\tau^{*}$. Therefore, a bad selection of the parameters will directly impact the equivalence between the original system and the reconstructed phase space. Thus, some authors are in favor of jointly selecting *p* and $\tau^{*}$ as, for example, the small-window solution [10], C–C method [11], and automated embedding [12].

Many researchers consider that the second approach (joint selection) is more reasonable than the first one (independent selection) in the engineering practice. They consider that the estimation of mutual information is rather cumbersome computationally, whereas the autocorrelation function only accounts for the linear dependence and therefore does not properly treat the presence of nonlinearities. On the other hand, the C-C method suggested by Kim et al. [11] is the most popular, which provides the delay time $\tau^{*}$ and embedding dimension *p* simultaneously by using correlation integral, and it has the advantage of low complexity and robustness in finite samples [13].

In the present paper, we propose a new method for selection *p* and $\tau^{*}$ based on symbolic dynamics and Information Theory. Symbolic Dynamics studies dynamical systems on the basis of the symbol sequences obtained for a suitable partition of the state space. The basic idea behind symbolic dynamics is to divide the phase space into finite number of regions and label each region by an alphabetical letter. In this regard, symbolic dynamics is a coarse-grained description of dynamics. Even though coarse-grained methods lose a certain amount of detailed information, some essential features of the dynamics may be kept, e.g., periodicities and dependencies, among others. Symbolic dynamics has been used for investigation of nonlinear dynamical systems (central references for the interested reader are [14,15,16,17,18]; for an overview, see Hao and Zheng [19]). In general terms, there is a broad agreement in that symbolization can increase the efficiency of finding and even quantifying information for characterizing and recognizing temporal patterns (see [20] for a review on experimental data). The process of symbolizing a time series is based upon the method of delay time coordinates, introduced by Takens, in order to carry out the phase space reconstruction.

Since the methods of state space reconstruction are based to some extent on detection of delays for which there is some sort of dependence (linear or nonlinear), and Symbolic Dynamics has been used as a statistical tool to detect the presence of dependence in time series [21]; symbolic dynamics is a suitable tool to select the optimal state space reconstruction parameters of chaotic time series.

Thus, we will select *p* and $\tau^{*}$ by translating the problem into symbolic dynamics and then we use a entropy measure associated with the symbols space (symbolic entropy) as a tool for the parameter selection. On the one hand, we have compared the performance of the proposed method with other available methods. Results seem to be in favor of this proposal. On the other hand, and from an empirical point of view, we have applied it to EEG data, which allows for understanding some dynamic characteristics of brain activity under epileptic episodes.

The rest of the paper is structured as follows: in Section 2, we introduce the basic concepts of symbolic analysis, and we also provide a symbolization procedure that works for estimation of the parameters for Phase Space reconstruction. In Section 3, we show the performance of the symbolic method to estimate phase space reconstruction parameters, and we compare it with the well known Mutual Information based methods and C-C method. In Section 4, the new techniques presented in this paper are applied to a real EEG database obtained from the University of Bonn and studied well for understanding epileptic phenomena. Finally, Section 5 presents conclusions.

## 2. Definitions and Symbolization Procedure

In this section, we will introduce some definitions and basic notations referred to symbolic dynamics.

Let ${\left\{X_{t}\right\}}_{t\in I}$ be a real valued time series. We will use symbolic analysis to study the state space reconstruction parameters associated with it. Symbolic analysis in our context is a coarse-grained approach to the study of time series, consisting of embedding the time series in a *p*-dimensional space, then constructing a partition of this *p*-dimensional space and labeling each set of the partition with a symbol. Therefore, all the *p*-dimensional vectors belonging to the same set of the partition are labelled with the same symbol. Afterwards, with information theory based measures, we will study the distribution of the symbols that will help us in the estimation of the parameters for state space reconstruction.

More concretely, in mathematical terms, given a positive integer p≥2, and a time delay τ, we consider that the time series is embedded in an *p*-dimensional space as follows:

(1) $$X_{p}^{\tau }\left(t\right)=\left(X_{t},X_{t+\tau },...,X_{t+(p-1)\tau }\right).$$

The parameter *p* is usually known as embedding dimension and $X_{p}^{\tau }\left(t\right)$p,τ-history.

Next, given a positive real number ϵ and in order to provide a partition of $R^{p}$, we define for any element $v=\left(v_{1},v_{2},\cdots ,v_{p}\right)\in R^{p}$ the following indicator function:

(2) $$\delta_{ij}\left(v\right)=\left\{\begin{array}{cc} 1 & \;\mathrm{if}\;|v_{i}\left|,\right|v_{j}\left|<\epsilon \;or\;\right|v_{i}\left|,\right|v_{j}|\geq \epsilon \\ 0 & \;\mathrm{otherwise}.\; \end{array}\right.$$

That is, $\delta_{ij}\left(v\right)=1$ if and only if its entries $v_{i}$ and $v_{j}$ satisfy that $|v_{i}|$ and $|v_{j}|$ are both either smaller or greater than ϵ. Let the set $\Gamma_{p}$ be the set of cardinality $2^{p-1}$ formed by the vectors of length p−1 with entries in the set {0,1}. Then, we can define a map $f_{\epsilon }:R^{p}⟶\Gamma_{p}$ defined as

$$f_{\epsilon }\left(v=\left(v_{1},v_{2},\cdots ,v_{p}\right)\right)=\left(\delta_{12}\left(v\right),\delta_{13}\left(v\right),\cdots ,\delta_{1p}\left(v\right)\right).$$

The map $f_{\epsilon }$ defines an equivalence relation in $R^{p}$ such that $v_{1}\;v_{2}$ if and only if $f_{\epsilon }\left(v_{1}\right)=f_{\epsilon }\left(v_{2}\right)$. Therefore, this equivalence relation provides a partition of $R^{p}$ in $2^{p-1}$ disjoint sets. Each of these sets is labeled with an element of $\Gamma_{p}$. The elements in $\Gamma_{p}$ are called symbols and $f_{\epsilon }$ symbolization map. In general, if $\pi \in \Gamma_{p}$ is a symbol and $v\in R^{p}$ is such that $f_{\epsilon }\left(v\right)=\pi$, then we will say that *v* is of π-type.

Next, we are interested in the application of the symbolization map $f_{\epsilon }$ to the p,τ-history $X_{p}^{\tau }\left(t\right)=\left(X_{t},X_{t+\tau },...,X_{t+(p-1)\tau }\right).$ Notice that $f_{\epsilon }\left(X_{p}^{\tau }\left(t\right)\right)$ is a vector (symbol) whose *i*-th entry provides information on whether $|X_{t}|$ and $|X_{t+i}|$ are both either smaller or greater than ϵ. Then, we want to extract information on the dynamics of the time series ${\left\{X_{t}\right\}}_{t\in I}$ by using information theory based measures on its associated symbols distribution ${\left\{f_{\epsilon }\left(X_{p}^{\tau }\left(t\right)\right)\right\}}_{t=1}^{T-(p-1)\tau }$. More concretely, we can estimate the probability of a symbol $\pi \in \Gamma_{p}$ as

(3) $$p_{\pi }=\frac{\#\;\{X_{p}^{\tau }\left(t\right)of\;\pi -type\}}{T-(p-1)\tau }$$

Now, under this setting, given a time delay τ and embedding dimension p≥2, we can define the symbolic entropy of a time process ${\left\{X_{t}\right\}}_{t\in I}$. This entropy is defined as the Shannon’s entropy [22] of the $2^{p-1}$ distinct symbols as follows:

(4) $$h\left(p,\tau \right)=-\sum_{\pi \in \Gamma_{m}}p_{\pi }\ln\left(p_{\pi }\right).$$

Symbolic entropy h(p,τ) is the information contained in comparing p,τ-histories generated by the time process. Notice that 0≤h(p,τ)≤ln(n), where the lower bound is attained when only one symbol occurs, and the upper bound for a completely random system (i.i.d temporal sequence) where all possible symbols appear with the same probability.

Then, if $\tau =\tau^{*}$ is an optimal time delay, for a positive integer *k*, the dependence between $X_{t}$ and $X_{t+k\tau^{*}}$ vanishes, and hence the symbolic entropy associated with the time series $\left\{X_{t}\right\}$ should be maximum. Therefore, in order to select the optimal time delay $\tau^{*}$, we select the first τ satisfying

(5) $$\tau^{*}=\arg\;\max_{\tau }\left\{h\left(p,\tau \right)\right\}$$

With respect to the optimal embedding window $\tau_{w}=\left(p-1\right)\tau^{*}$, this can be associated with the mean orbital period $P_{w}$ of low-dimensional chaotic systems that shows pseudo-periodicity. That is, $P_{w}$ can be considered the time dependence of the chaotic time series. Although the chaotic systems oscillate without periodicity, low dimensional chaotic systems show pseudo-periodicity. The mean orbital period could naturally be associated with the mean time between two consecutive visits to a Poincare section [23]. For the time series with mean orbital period $P_{w}$, all points at a time multiple of $P_{w}$ are in the same Poincare section in phase space. Therefore, a local minimum of symbolic entropy h(p,τ) is reached for $\tau =kP_{w}$ and thus

(6) $$\tau_{w}=\arg\min_{\tau }\left\{h\left(p,\tau \right)\right\}.$$

To finish this section, we are going to illustrate the symbolization procedure with an easy example. Let ${\left\{X_{t}\right\}}_{t\in I}$ be the following finite time series:

(7) $$\left\{X_{1}=2;X_{2}=-7;X_{3}=-12;X_{4}=5;X_{5}=-1;X_{6}=9;X_{7}=14\right\}$$

and assume that ϵ=3, τ=1 and p=3. Then, the symbols’ set remains as

$$\Gamma_{3}=\left\{\left(0,0\right);\left(0,1\right);\left(1,0\right);\left(1,1\right)\right\}.$$

Under this setting, we can construct the following five p,τ-histories: $X_{3}^{1}\left(1\right)=\left(1,-7,-12\right)$; $X_{3}^{1}\left(2\right)=\left(-7,-12,5\right)$; $X_{3}^{1}\left(3\right)=\left(12,5,-1\right)$; $X_{3}^{1}\left(4\right)=\left(5,-1,9\right)$; and $X_{3}^{1}\left(5\right)=\left(-1,9,14\right)$. Then, the symbolization map $f_{\epsilon }$ associate each p,τ-history to a symbol. Concretely, $f_{3}\left(X_{3}^{1}\left(1\right)=\left(1,-7,-12\right)\right)=\left(0,0\right)$ because the first entry of the m-history, 1 is in absolute value smaller than ϵ=3 while the second and the third are both greater than ϵ=3, and hence the agreement indicator that defines the symbolization map takes the value 0. Similarly, we find that $X_{3}^{1}\left(2\right)$ is of (1,1)−type, $X_{3}^{1}\left(3\right)$ is of (1,0)−type, $X_{3}^{1}\left(4\right)$ is of (0,1)−type, and $X_{3}^{1}\left(5\right)$ is of (0,0)−type. Thus, we can estimate the symbols distribution by its relative frequency $p\left(\left(0,0\right)\right)=\frac{2}{5}$, $p\left(\left(0,1\right)\right)=p\left(\left(1,0\right)\right)=p\left(\left(1,1\right)\right)=\frac{1}{5}$ and the entropy associated with them $h\left(p,\tau \right)=h\left(3,1\right)=-\frac{2}{5}\log\left(\frac{2}{5}\right)-\frac{1}{5}\log\left(\frac{1}{5}\right)-\frac{1}{5}\log\left(\frac{1}{5}\right)-\frac{1}{5}\log\left(\frac{1}{5}\right)=1.3322$.

### Selection of *p* and ϵ for Finite Sample Sizes

When determining the parameters of phase space reconstruction of a finite chaotic time series by using the symbolic entropy, one needs to select in advance the values of *p* and ϵ. In addition, sample size *T* also plays an important role. In [21], some general criteria are recommended to select the embedding dimension *p* and sample size *T* in order to compute the symbolic entropy. First, the sample size *T* should be as larger than the number of symbols $2^{p-1}$ of the symbolization map $f_{\epsilon }$. Second, from a statistical point of view, data sets must contain at least five times the number of possible events or symbols. Thus, the embedding dimension will be the largest positive integer *p* that satisfies $5\cdot 2^{p-1}\leq T$.

To select ϵ, we propose to use a data driven method which is based on symbolic entropy. Particularly, we partially rely on the methodology described in [24] based on the construction of peak detection functions (FPs). The selected ϵ will be the largest ϵ that locally maximizes the absolute value of a pick function $FP(i,x_{i})$, where FP associates values to the symbolic entropy of a time series. More concretely, define

(8) $$FP^{+}\left(k,\tau \right)=\frac{\max\left\{h_{\tau }-h_{\tau -1},\;h_{\tau }-h_{\tau -2},...,\;h_{\tau }-h_{\tau -k}\right\}+\max\left\{h_{\tau }-h_{\tau +1},\;h_{\tau }-h_{\tau +2},...,\;h_{\tau }-h_{\tau +k}\right\}}{2}$$

and

(9) $$FP^{-}\left(k,\tau \right)=\frac{\min\left\{h_{\tau }-h_{\tau -1},\;h_{\tau }-h_{\tau -2},...,\;h_{\tau }-h_{\tau -k}\right\}+\min\left\{h_{\tau }-h_{\tau +1},\;h_{\tau }-h_{\tau +2},...,\;h_{\tau }-h_{\tau +k}\right\}}{2}$$

where $h_{\tau +l}=h\left(p,\tau +l\right)$ for l=0,1,..k. The functions $FP^{+}\left(k,\tau \right)$ (respectively $FP^{-}\left(k,\tau \right)$) allows for selecting the time delay τ for which value h(p,τ) is maximum (respectively minimum) in the neighborhood of (τ−k,τ+k). As stated in [24], values of *k* in the range [3,5] are usually suitable. Notice that, by construction, $0<\epsilon <\max\{X_{t}\}$. Then, the selected parameter, namely $\epsilon^{*}$, will be the one in the interval $\left(0,\max\left\{X_{t}\right\}\right)$ that satisfies

(10) $$\epsilon^{*}=\max_{\epsilon }\{\max_{\tau }\left\{FP^{+}\left(k,\tau \right)\right\},\min_{\tau }\{|FP^{-}\left(k,\tau \right)|\}\}$$

## 3. Simulation Analysis

The following examples illustrate the performance of the proposed symbolic method when estimating the parameters time delay $\tau^{*}$ and embedding dimension *p* for phase space reconstruction of a chaotic time series. The aim of this set of simulations is, firstly, to empirically evaluate the performance of the new symbolic procedure to select the “correct” parameters. Secondly, we aim to compare with the symbolic method with other competitive available methods that have been commented in the introductory section and that are fully documented in the bibliographical references of this paper.

To this end, we extract univariate time series $\left\{X_{t}\right\}$ of length T=3000 from five chaotic systems that have been extensively studied. In all cases, we set the embedded time series in a six-dimensional space that is p=6. To evaluate the performance of the novel symbolic method, we compare the performances of the new method with other available selection methods: the C-C method (C-C), the Nearest Neighbor method, and the method based on the first minimum of the autocorrelation function (FAC) selection parameters of these last two methods are based on the Mutual Information (MI) criteria.

Scientific literature has shown that the C-C method has a good performance when used for selecting time delays and embedding dimensions. Thus, the C-C method can be thought of as a natural competitor and therefore it is worth comparing the performance against it. For this reason, we will compare results for several well-known dynamic systems. In order to compare and evaluate the performance of each method, we will use the selected parameters of each method for reconstructing the attractor and estimating two complexity measures of each system. These two measures are theoretically known for each of the three systems, and therefore they are used as a base of comparison. A final user will prefer using reconstruction parameters that lead to estimations that are as close as possible to the theoretical ones.

**Figure 1:**
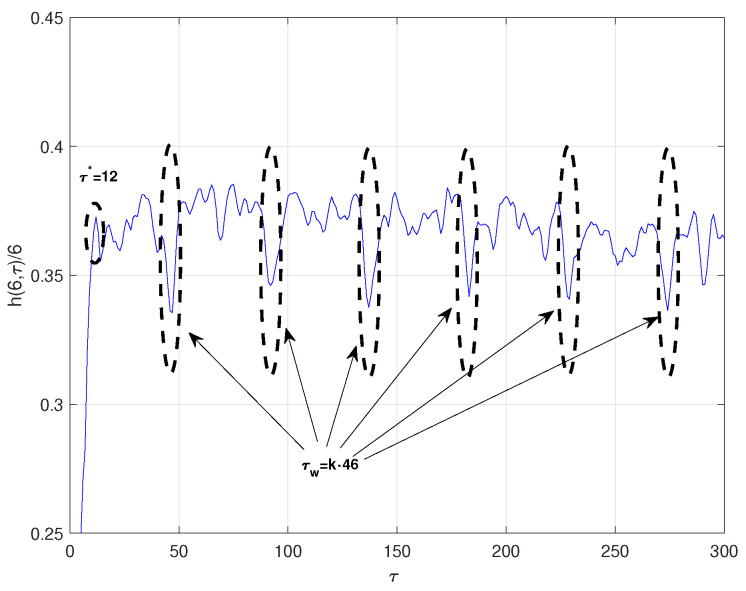
Normalized symbolic entropy h(6,τ)/6 for $\epsilon =1.2\sigma_{x}$ of Lorenz system.

**Figure 2:**
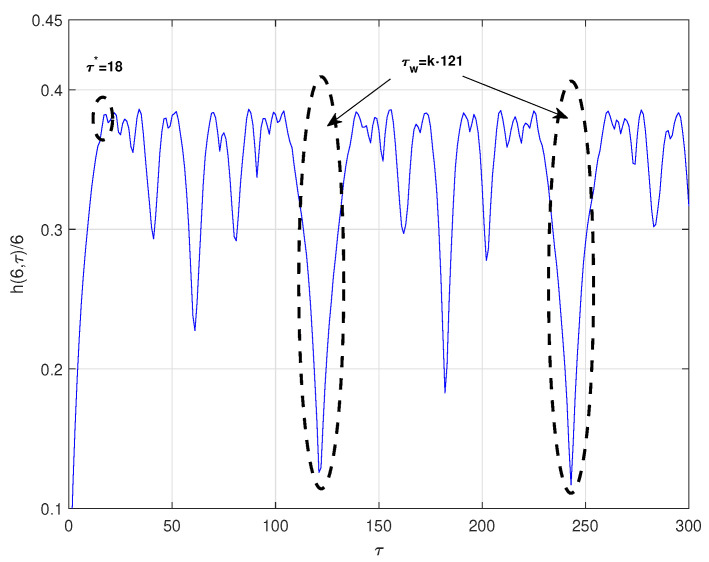
Normalized symbolic entropy h(6,τ)/6 for $\epsilon =0.4\sigma_{x}$ of Rossler system.

**Figure 3:**
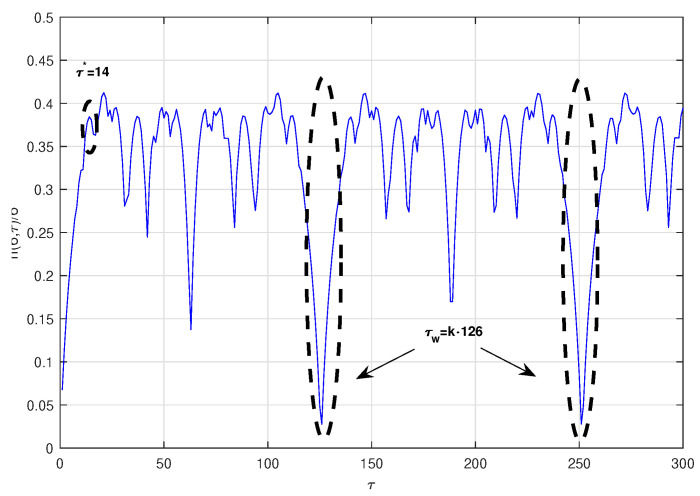
Normalized symbolic entropy h(6,τ)/6 for $\epsilon =0.275\sigma_{x}$ of the Duffing system.

**Figure 4:**
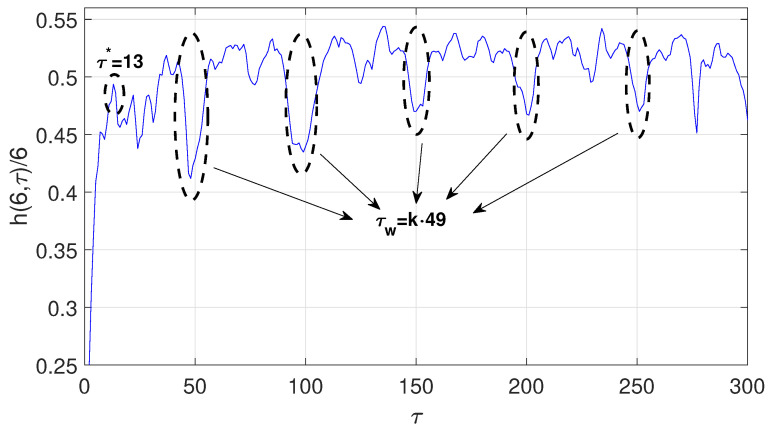
h(6,τ)/6 for $\epsilon =0.79\sigma_{x}$ of the Mackey–Glass system.

**Figure 5:**
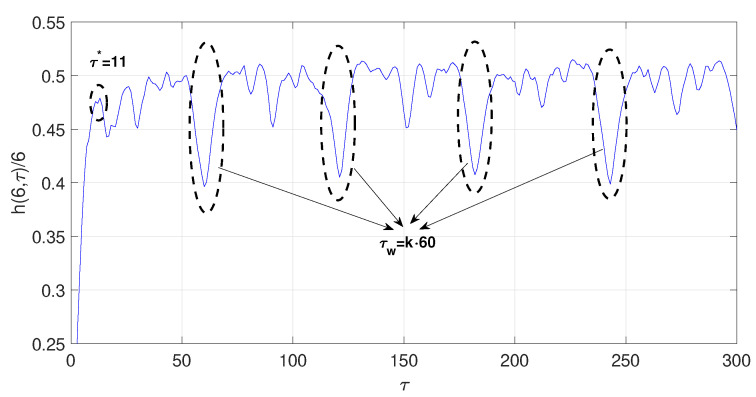
Normalized symbolic entropy h(6,τ)/6 for $\epsilon =0.89\sigma_{x}$ of the Chen system.

Accordingly, we will use the following systems to conduct the comparisons:

- Lorenz system [25]:
  (11) $$\begin{array}{ccc} \dot{x} & = & -a(x-y)\\ \dot{y} & = & -xz+cx-y\\ \dot{z} & = & xy-bz \end{array}$$
  The time series was obtained by projecting the *x*-coordinate of the system defined by the parameters *a* = 16, *c* = 45.92, *b* = 4, with an integral step 0.01, and initial conditions $x_{0}=-1$, $y_{0}=0$ y $z_{0}=1$. The computed optimal ratio is $\epsilon^{*}=1.2\sigma_{x}$, where $\sigma_{x}$ is the standard deviation of the chaotic time series under consideration. For this optimal radio, Figure 1 illustrates the series of the normalized symbolic entropy h(6,τ)/6 as a function of the time delay τ for the Lorenz system. Clearly, we observe that the first local maximum is attained at $\tau^{*}=12$ and the minimum at $\tau_{w}=46$. Then, an estimated value of embedding dimension *p* can be computed by solving $\tau_{w}=\left(p-1\right)\tau^{*}$ obtaining an approximate value of p=5. For the Mutual Information method, the optimal time delay was $\tau^{*}=11$, while, for the C-C method, the estimated parameters were $\tau^{*}=10$$\tau_{w}=100$ and p=11. Notice that the optimal time delay $\tau^{*}$ estimated by the three methods are quite close to each other while the estimated time delay window $\tau_{w}$ strongly differs between C-C and symbolic methods:
- Rossler system [26]:
  (12) $$\begin{array}{ccc} \dot{x} & = & -y-z\\ \dot{y} & = & x+dy\\ \dot{x} & = & z(x-f)+e \end{array}$$
  The time series was obtained by projecting the *x*-coordinate of the system defined by the parameters d=0.15, e=0.2 and f=10, with an integral step 0.05, and initial conditions $x_{0}=-1$, $y_{0}=0$ and $z_{0}=1$. The computed optimal radio is $\epsilon^{*}=0.4\sigma_{x}$, where $\sigma_{x}$ is the standard deviation of the chaotic time series under consideration. Figure 2 shows the normalized symbolic entropy h(6,τ)/6 as a function of the time delay τ for the Rossler system. It can be seen that the selected parameters by the symbolic method are $\tau^{*}=18$ and $\tau_{w}=121$, and consequently the estimated value for *p* is 8. The estimated time delay for Mutual Information method is $\tau^{*}=20$. For the C-C method, the estimated parameters are $\tau^{*}=17$ and $\tau_{w}=191$. Again, the optimal time delay $\tau^{*}$ estimated by the three methods is very similar while the time delay window $\tau_{w}$ estimated by the C-C method is much different than the one estimated with symbolic entropy.
- Duffing System [27]:
  (13) $$\begin{array}{ccc} \dot{x} & = & y\\ \dot{y} & = & -gy-kx(1+x^{2})+lcosz\\ \dot{z} & = & v \end{array}$$
  The time series was obtained by projecting the *x*-coordinate of the system defined by the parameters g=0.05, k=0.25, l=7.5 and v=1, with an integral step 0.05, and initial conditions $x_{0}=-1$, $y_{0}=0$ y $z_{0}=1$. The computed optimal radio is $\epsilon^{*}=0.275\sigma_{x}$, where $\sigma_{x}$ is the standard deviation of the chaotic time series under consideration. Figure 3 shows the normalized symbolic entropy h(6,τ)/6 as a function of the time delay τ for the Duffing system. The estimated optimal time delay and time delay window with the symbolic method are $\tau^{*}=14$ and $\tau_{w}=126$, respectively. Then, the estimated embedding dimension is p=10. As in the previous examples, the estimated time delay for the Mutual Information method ($\tau^{*}=12$) and for the C-C method ($\tau^{*}=12$) are fairly close to the one estimated by symbolic method. Again, the time delay window estimated based on the C-C method $\tau_{w}=161$ is far from the one estimated with symbolic method.
  These first three models are well-known and well-studied and have served as a base of comparison of new techniques for similar aims as this paper. In order to complete this analysis, we have also considered the next two models that we refer to as the Mackey–Glass model and Chen model:
- Mackey-Glass system [28]:
  (14) $$\frac{dx}{dt}=\frac{ax(t-\tau )}{1+x^{c}\left(t-\tau \right)}-bx$$
  The time series was obtained by fixing parameters a = 0;2, b = 0;1, c = 10, y = 17, with initial conditions x(t < 0) = 0 y x(t = 0) = 1;2. The first 2000 iterations were discarded. The computed optimal radio is $\epsilon^{*}=0.79\sigma_{x}$, where $\sigma_{x}$ is the standard deviation of the chaotic time series under consideration. The estimated optimal time delay and time delay window with the symbolic method are $\tau^{*}=13$ and $\tau_{w}=49$, respectively. Figure 4 shows the normalized symbolic entropy h(6,τ)/6 as a function of the time delay τ for the Mackey-Glass system Then, the estimated embedding dimension is p=5. In this case, the estimated time delay for the Mutual Information method ($\tau^{*}=12$) and for the C-C method ($\tau^{*}=14$) are fairly close to the one estimated by symbolic method (τ=13). Again, the time delay window estimated based on the C-C method $\tau_{w}=166$ is far from the one estimated with symbolic method($\tau_{w}=49$):
- Chen system [29]:
  $$\begin{array}{ccc} \dot{x} & = & a(y-x)\\ \dot{y} & = & (c-a)x+cy-xz\\ \dot{z} & = & xy-bz \end{array}$$
  The time series was obtained by projecting the *x*-coordinate of the system defined by the parameters a=35,b=3,c=28, with an integral step 0.01, and initial conditions $x_{0}=-1$, $y_{0}=0$ y $z_{0}=1$. The first 2000 iterations were discarded. The computed optimal radio is $\epsilon^{*}=0.89\sigma_{x}$, where $\sigma_{x}$ is the standard deviation of the chaotic time series under consideration. The estimated optimal time delay and time delay window with the symbolic method are $\tau^{*}=11$ and $\tau_{w}=60$, respectively. Figure 5 shows the normalized symbolic entropy h(6,τ)/6 as a function of the time delay τ for the Chen system. Then, the estimated embedding dimension is p=6. As in the previous examples, the estimated time delay for the Mutual Information method ($\tau^{*}=10$) and for the C-C method ($\tau^{*}=9$) are fairly close to the one estimated by symbolic method. Again, the time delay window estimated based on the C-C method $\tau_{w}=104$ is far from the one estimated with symbolic method ($\tau_{w}=60$).

Table 1 summarizes for each method the estimated parameters for phase space reconstruction of the five systems. Bold is reserved for the results obtained with the new selecting method.

In order to check whether the symbolic method is reliable when estimating the parameters for phase space reconstruction, $\tau^{*}$, $\tau_{w}$, and *p*, we will compute, based on this estimation, two complexity measures for each one of the systems that needs these parameters for its computation. These complexity measures are the largest Lyapunov exponent LLE[30], which is a measure of the complexity of the time process, and the Correlation Dimension D[31], which is a measure of the dimension of the space occupied by the chaotic attractor. For the computation of these two geometric invariants, the time delay $\tau^{*}$ and embedding dimension *p* are essential parameters, and a bad selection of them would produce a big bias in LLE and D. The largest Lyapunov exponent LLE for the five systems have been computed in [27,32,33,34,35] and the Correlation Dimension D in [32,33,36,37]. Furthermore, we have completed the study by increasing the sample size to 10,000 observations. Table 2 and Table 3 show the values of LLE and D based on the values of the estimated parameters $\tau^{*}$, $\tau_{w}$ and *p* with symbolic and C−C methods together with the reference values, respectively.

We can observe the estimated values of the largest Lyapunov exponent and Correlation dimension based on the Symbolic method in Table 2 and Table 3, respectively. Importantly, these symbolic-based estimations are very close to their reference (theoretical) values, regardless of the sample size, which suggests the good behavior of the new method for reconstruction of the dynamics of the system. On the other hand, we were wondering if the symbolic method is competitive with its main competitor, namely, the C-C method. In this regard, we can devise that the estimated values for the Lyapunov Exponents are clearly in favor of the Symbolic method as the estimation is closer to the theoretical reference value than in the case of the C-C method. This is true for the five systems. Similar conclusions can be obtained from the results regarding correlation dimension: the symbolic-based estimated dimensions are closer to the true value than C-C estimation, regardless the studied system. On the other hand, methods based on nearest neighbors and autocorrelation function are reported. Results show the symbolic based method also has better empirical behavior. All of these results could be explained by a wrong selection of delay time window $\tau_{w}$ by the C-C method as stated in [23,38,39,40].

## 4. EEG Dynamics under Epileptic Activity

The Electroencephalogram (EEG) is a spontaneous bioelectricity activity that is produced by the central nervous system. Therefore, EEG can be understood as a representative signal containing information about the activity of the brain. Currently, EEG is widely used in clinic and neuralelectricity physiological research. The shape of the waves may contain useful information about the state of the brain. EEG does include abundant information about the state and change of the neural system.

The dynamics of brain activity is considered to be of a nonlinear nature. Accordingly, EEG signals are studied by means of nonlinear dynamic tools. Indeed, a large body of studies have reported that the EEG was derived from chaotic systems [41,42,43,44].

In this section of the paper, we apply the symbolic-based approach for reconstruction of dynamics generated by empirical EEG recording from a public dataset by the University of Bonn [41]. Epilepsy is characterized by recurring seizures in which abnormal electrical activity in the brain causes the loss of consciousness or a whole body convulsion. From this point of view, our results will contribute to the empirical analysis of role on nonlinear dynamics in epileptology. The Bonn University EEG database is comprised of five types of EEG signals (EEG recordings from healthy volunteer with eyes open and closed, epilepsy patients in the epileptogenic zone during a seizure-free interval and in an opposite brain zone, and epilepsy patients during epileptic seizures) were studied.

To conduct this empirical analysis, we firstly use Theiler’s method of surrogate data to distinguish between linearity and nonlinearity. To do so, the null hypothesis of linearity is tested against nonlinearity [45]. Chaos cannot come from a linear signal. Secondly, we test for chaoticity against pure stochasticity. Linear signals are expected to be of stochastic nature while nonlinear signals can come from either a stochastic process or a pure chaotic one. The statistical test for chaos [46] tests the null hypothesis of chaos versus the alternative of stochastic process. We also estimate correlation dimension using Theiler’s approach in order to exclude time correlated states in the correlation integral estimation [47].

Table 4 collects the outcomes of all procedures. Results firstly indicate that the brain’s activity is of a nonlinear nature for a healthy person with open eyes and for records of epileptic person regardless if s/he is under seizure activity or not, whenever measurement is done in the epileptogenic zone. The test for chaos applied to nonlinear signals helps to conclude that only the nonlinear dynamics found for epileptic patients are statistically compatible with chaotic dynamics, while the dynamics are nonlinear stochastic for a healthy person with open eyes. Finally, the estimated correlation dimensions show how (correlation) dimension is reduced as the process moves from stochastic to chaotic, as expected.

These results support the nonlinear deterministic structure of brain dynamics related to epileptic activity as earlier reported in [48,49]. Our estimates of correlation dimensions are in line with other previous studies [50] on the same dataset, although with different parameter configurations. Thus, the conclusion in this regard is that epileptic seizures are emergent states with reduced dimensionality compared to non-epileptic activity. This is in line with the clinical common knowledge that establishes that healthy systems evolve with time and their adaptive capability is higher, resulting in higher complexity. On the other hand, the alternations in the structural components and/or decreased functional capability of the subsystem cause dysfunction in the regularity mechanism of the overall system, which results in the loss of complexity, as indicated in [51,52].

## 5. Conclusions

In this paper, we have introduced a new method based in Symbolic Dynamics, for the estimation of the phase space reconstruction parameters $\tau^{*}$, $\tau_{w}$ and *p*. In the simulation analysis, we applied the Symbolic method to choose the phase space reconstruction parameters from the time series generated from several dynamical models that have been well-studied and used for evaluating the ability of different reconstruction methodologies. The values found for $\tau^{*}$ agree well with those found for the mutual information and the C-C method. The values found for $\tau_{w}$ do not agree with the values estimated by the C-C method. For this reason, in order to clarify which method for selecting phase space reconstruction parameters is more reliable, we use them in the computation of two complexity measures, namely largest Lyapunov exponent (LLE) and Correlation dimension (D). Results indicate that the parameters estimated by the Symbolic method produces a closer approach to reference (theoretical) values of LLE and D than the C-C method. Finally, the proposed method is used to study the dynamics of brain activity under epilepsy by means of real EEG signals. The empirical results hint that epileptic patients show chaotic dynamics in EEG signals. Furthermore, our results are statistically significant and therefore hint the potential of symbolic based tools in distinguishing healthy and epileptic subjects.

## Figures and Tables

**Table 1:** Estimated parameters for phase space reconstruction for the studied systems.

	MI Method	C-C Method			Symbolic Method			NN		FAC	
System	$\tau^{*}$	$\tau^{*}$	$\tau_{w}$	*p*	$\tau^{*}$	$\tau_{w}$	*p*	$\tau^{*}$	*p*	$\tau^{*}$	*p*
Lorenz	11	10	100	11	**12**	**46**	**5**	11	3	11	3
Rossler	20	17	191	12	**18**	**121**	**8**	20	4	20	4
Duffing	12	14	161	12	**14**	**126**	**10**	12	2	12	2
Mc-Glass	12	14	166	13	**13**	**49**	**5**	12	4	12	4
Chen	10	9	104	27	**11**	**60**	**6**	10	4	10	4

**Table 2:** Largest Lyapunov exponent LLE based on the estimation of the phase space parameters $\tau^{*}$, $\tau_{w}$ and *p* if C-C method and symbolic method, together with the reference true value. Values in parentheses report estimated LLE for series of 10,000 observations.

	LLE Reference Value	Estimated LLE C-C Method	Estimated LLE Symbolic Method	Estimated LLE NN	Estimated LLE FAC
Lorenz	1.500	0.940 (1.667)	**1.50 (1.670)**	1.438 (1.659)	0.771 (0.742)
Rossler	0.090	0.095 (0.066)	**0.09 (0.080)**	0.061 (0.079)	1.108 (0.068)
Duffing	0.183	0.168 (0.021)	**0.184 (0.200)**	0.014 (0.215)	0.019 (0.02)
Mc-Glass	0.007	0.006 (0.008)	**0.007 (0.007)**	0.006 (0.009)	0.007 (0.008)
Chen	1.997	1.852 (1.773)	**1.982 (1.852)**	2.483 (2.160)	1.359 (1.120)

**Table 3:** Correlation dimension D based on the estimation of the phase space parameters $\tau^{*}$, $\tau_{w}$, and *p* of the C-C method and symbolic method, together with the reference true value. Values in parentheses report estimated *D* for series of 10,000 observations.

	D Reference Value	Estimated D C-C Method	Estimated D Symbolic Method	Estimated D NN	Estimated D FAC
Lorenz	2.06	1.93 (2.41)	**2.01 (2.02)**	1.76 (2.23)	2.02 (3.63)
Rossler	2.01	2.13 (2.31)	**2.09 (2.01)**	1.79 (1.77)	1.92 (1.49)
Duffing	2.23	2.10 (2.56)	**2.21 (2.37)**	1.13 (1.16)	1.51 (1.47)
Mc-Glass	2.10	2.83 (2.20)	**2.12 (2.11)**	1.94 (1.80)	2.01 (1.97)
Chen	2.16	2.19 (3.67)	**2.13 (2.12)**	2.05 (2.26)	2.36 (3.49)

**Table 4:** Complexity measures for EEG data sets.

Brain Status	$\tau^{*}$	$\tau_{w}$	*p*	Non Linearity Test	Chaos vs. Stochastic	Estimated D
Healthy with open eyes	13	98	9	NL (pval=0.03)	Stochastic(pval<0.001)	5.56
Healthy with closed eyes	9	109	13	L (pval=0.64)	-	7.98
Seizure free non-epileptogenic zone	9	120	14	L (pval=0.76)	-	4.99
Seizure free epileptogenic zone	18	108	7	NL (pval<0.001)	Chaos(pval=1)	3.48
Seizure activity	11	65	7	NL (pval<0.001)	Chaos(pval=1)	3.69
